# Supplementary material for: Incidence and predictors of loss to follow up among adult HIV patients on antiretroviral therapy in University of Gondar Comprehensive Specialized Hospital: A competing risk regression modeling
Source: PLoS One. 2020 Jan 24;15(1):e0227473. doi: 10.1371/journal.pone.0227473 (PMC6980595; doi:10.1371/journal.pone.0227473)
Supplement: Attachment — Submitted filename: response to editors.docx [file pone.0227473.s002.docx]

**Dear PLoS one editors**

Thank you for your valuable comments for the betterment of our paper or manuscript.

Here are the comments raised by your editorial staff and Authors response

**Comments you raised**

Our editorial staff has assessed your submission, and we have significant concerns about the grammar, usage, and overall readability of the manuscript. We believe that the manuscript is much more likely to be accepted if it is easy for the reviewers to read and understand. We, therefore, request that you revise the text to fix the grammatical errors and improve the overall readability of the text before we send it for review.

**Authors response**

After consulting language experts who had an MA degree in TEAFL (teaching English as a foreign language) and who had many years' experience in the area of literature at the University of Gondar, we corrected all the comments and we attached the revised version of our manuscript.
